# Protection of NOx Sensors from Sulfur Poisoning in Glass Furnaces by the Optimization of a “SO_2_ Trap”

**DOI:** 10.3390/s23198186

**Published:** 2023-09-30

**Authors:** Carole Naddour, Mathilde Rieu, Antoinette Boreave, Sonia Gil, Philippe Vernoux, Jean-Paul Viricelle

**Affiliations:** 1 Mines Saint-Etienne, Univ Lyon, CNRS, UMR 5307 LGF, Centre SPIN, F-42023 Saint-Etienne, France; carole.naddour@emse.fr (C.N.); viricelle@emse.fr (J.-P.V.); 2 Univ Lyon, Université Lyon 1, CNRS, UMR 5256, IRCELYON, 2 avenue Albert Einstein, F-69622 Villeurbanne, France; antoinette.boreave@ircelyon.univ-lyon1.fr (A.B.); sonia.gil@ircelyon.univ-lyon1.fr (S.G.); philippe.vernoux@ircelyon.univ-lyon1.fr (P.V.)

**Keywords:** NOx sensor, glass furnaces, sulfur poisoning, SO_2_ trap, CuO/BaO-based traps

## Abstract

Electrochemical NOx sensors based on yttria-stabilized zirconia (YSZ) provide a reliable onboard way to control NOx emissions from glass-melting furnaces. The main limitation is the poisoning of this sensor by sulfur oxides (SOx) contained in the stream. To overcome this drawback, an “SO_2_ trap” with high SOx storage capacity and low affinity to NOx is required. Two CuO/BaO/SBA-15 traps with the same CuO loading (6.5 wt.%) and different BaO loadings (5 and 24.5 wt.%, respectively) were synthetized, thoroughly characterized and evaluated as SO_2_ traps. The results show that the 6.5%CuO/5%BaO/SBA-15 trap displays the highest SO_2_ adsorption capacity and can fully adsorb SO_2_ for a specific period of time, while additionally displaying a very low NO adsorption capacity. A suitable quantity of this material located upstream of the sensor could provide total protection of the NOx sensor against sulfur poisoning in glass-furnace exhausts.

## 1. Introduction

Nitrous oxides (NOx) are well known as extremely harmful pollutants that play key roles in the generation of acid rain and photochemical smog and cause damage to human health [1]. In glass-melting furnaces, the combustion of natural gas, heavy oils, and other fuel sources is used to melt raw materials at high process temperatures of up to 1500 °C, resulting in NOx emissions in the exhaust gas [2,3]. Many countries and organizations have imposed stringent emission regulations on glass furnaces to reduce their NOx pollution levels. In this respect, the development of in situ, cheap and robust techniques, like NOx sensors, becomes urgent for the onboard control of NOx emissions during the combustion processes [1,4]. Electrochemical sensors based on a solid electrolyte are a promising method for the detection of NOx. They have been extensively studied due to their high sensitivity, selectivity, and durability. In this paper, we will only focus on the potentiometric gas sensor based on yttria-stabilized zirconia (YSZ) solid electrolyte, as reported by many research groups [5,6,7,8,9,10,11]. For example, Ferlazzo et al. [12] developed an electrochemical biosensor based on YSZ for amino acid detection. Ando et al. [13] studied a capacitive biosensor using a YSZ functional layer for the quantification of ammonia. Hao et al. [14] used a YSZ-based potentiometric sensor with ZnGa_2_O_4_ and Pt electrodes for the detection of SO_2_ under harsh environments with a high sensitivity. The YSZ-based sensors are generally distinguished by their high temperature resistance and high chemical stability, in addition to good and reproducible sensing performance [15].

Usually, a platinum reference electrode and a gold sensing electrode are associated with the YSZ electrolyte. The signal of this sensor type (ΔV= V_Pt_ − V_Au_) in a complex gas environment is based on the development of a mixed potential on the sensing electrode, as described in previous studies [16,17,18].

One of the major challenges in NOx sensors is the cross-sensitivity to other pollutants such as CO, as well as the hydrocarbons present in the exhaust. In order to eliminate this interference with untargeted gases and to obtain a higher NOx selectivity, a solution proposed in the literature is to use a catalytic filter [19,20,21] on top of the sensing electrode. For instance, Gao et al. [6,22] have developed a NOx-selective Pt/YSZ/Au sensor by coating a catalytic filter consisting of 1.7–4.5 wt.% Pt dispersed on alumina directly on the sensing elements of the sensor. They reported that this filter can fully oxidize CO and hydrocarbons, and thereby remove their interference. In addition, the catalytic filter allows for the reaching of the NO/NO_2_ thermodynamic equilibrium, resulting in a similar response for either NO or NO_2_ at equal concentrations. Therefore, the sensor response depends only on the total NOx concentration, regardless of the NO/NO_2_ ratio arriving at the sensor, at a constant oxygen partial-pressure and temperature. Another way to obtain selective and sensitive sensors is to apply a polarization current. Some studies reported that the polarization of the sensing electrode can control the selectivity of the sensor towards NO or NO_2_ [23,24,25]. J.P. Viricelle et al. [26] have proposed a sensitive three-electrode sensor based on the selective electrochemical reduction of NO_2_. In this study, the YSZ-based electrochemical cell operates upon electric polarization between an Au working electrode and a Pt counter-electrode, resulting in selective NO_2_ detection at 400–550 °C. When the Au working electrode is negatively polarized, only reducible gas (NO_2_) can affect its potential (at constant oxygen partial pressure) and therefore be detected, which avoids interference from reductive gases such as CO hydrocarbons. To achieve a selective and sensitive response to NOx and not only NO_2_, the idea of GaO et al. [22] was to combine both the catalytic filter and the electrical polarization on a three-electrode sensor.

However, for glass-melting furnace applications, one of the main issues is the presence of sulfur oxides (SOx) in the stream, which can significantly poison the electrodes and the Pt/Al_2_O_3_ catalytic filter of the NOx sensor [27,28]. Therefore, this study aims to develop a sulfur oxides “trap” located up-stream from the sensor to protect the NOx sensors from sulfur poisoning. Such material must exhibit a good catalytic activity for the oxidation of SO_2_ to SO_3_ and a high SOx storage capacity into sulfates. Furthermore, the overall NOx concentration in the stream must not be altered, excluding the NOx storage on the trap material. A large variety of materials have been reported as potential SOx traps, and can be classified in different categories such as single-oxides materials (CaO, MgO, TiO_2_, MnO_2_, CeO_2_, Al_2_O_3_), oxides supported on porous silica or alumina-based materials (zeolites, mesoporous or clay minerals) and oxides supported on carbonaceous materials (activated carbon and carbon fibers) [29,30,31,32,33,34,35,36,37]. Al_2_O_3_-based oxides undergo sulfation leading to a loss in their textural properties [38] and present a specific surface area (SSA) in the order of 200 m²/g, which is not high enough for this application. The main drawback of activated carbons is their limited thermal stability [39]. On the other hand, organized mesoporous silica SBA-15 could be a suitable support, combining a high thermal stability with a chemical inertia towards SOx, which was shown to protect the mesoporous structure from collapsing [38,40]. Moreover, SBA-15 presents a high SSA and porous volume that can highly disperse the SOx-storage active phases [41,42]. Many studies are focused on SOx traps based on copper oxide (CuO) as an active phase [38,43,44,45,46,47,48,49]. Xue et al. [50] found that among a series of adsorbents (CuO, Zn/Mn type, Zn/Ti/Zr type,Zn/Co type, and Zn/Al type), the CuO-based adsorbents exhibited higher reactivity levels for low-temperature desulfurization. Indeed, this oxide exhibits a significant catalytic activity towards the oxidation of SO_2_ to SO_3_ and a good ability to store SO_3_ as CuSO_4_. In addition, CuO can be easily regenerated after the decomposition of CuSO_4_ at a relatively low temperature (<700 °C), thereby limiting the energy consumption and preventing the support deterioration. Furthermore, SO_2_ released during the regeneration step can be recovered as sulfuric acid or pure sulfur [51,52].

On the other hand, BaO is a well-known NOx storage compound involved in NOx storage/reduction (NSR) catalysts developed for the post-treatment of diesel vehicles [53,54,55]. However, its deactivation by sulfur species is well documented in the literature [27,28,56]. For example, the SO_2_ exposure of Pt/BaO/Al_2_O_3_ and Pt/BaCO_3_/Al_2_O_3_ catalysts, used as NOx traps, has resulted in the formation of surface and bulk sulfates on barium sites. 

Indeed, BaO has a high ability for sulfation under harsh environments, and the resulting barium sulfates are more stable than barium nitrates [55,56]. Moreover, since the storage capacity depends on the experimental conditions, especially on the reaction temperature, it seems possible to make the SOx adsorption predominant with respect to that of NOx on BaO sites. However, BaO is not active for the oxidation of SO_2_ to SO_3_ and cannot be used as the sole active phase in the catalyst. In this context, in addition to CuO, BaO was chosen as a second active phase to further increase the SOx storage capacity.

In this work, the SO_2_ storage capacity of two CuO/BaO/SBA-15 adsorbents with different BaO loadings were assessed under simulated gases from glass-furnace exhaust during multicycle experiments. These traps were characterized by the inductively coupled plasma method (ICP), X-ray diffraction (XRD), N_2_ physisorption, laser diffraction granulometry, and scanning electron microscopy (SEM). The performances of potentiometric NOx sensors were also investigated when coupled, or not, with an upstream SO_2_ trap. Moreover, the presence of sulfites/sulfate species on the most efficient adsorbent after several cycles of NO-SO_2_ exposures was confirmed by diffuse reflectance infrared Fourier transform spectroscopy (DRIFTS).

## 2. Materials and Methods

### 2.1. Synthesis of CuO/BaO/SBA-15 Traps

A commercial SBA-15 mesoporous silica (XFNANO) was first calcined at 900 °C for 2 h with a ramp of 10 °C/min in static air. The CuO/BaO/SBA-15 SO_2_ traps were prepared as follows. First, CuO was wet-impregnated on the pre-calcined SBA support: the targeted amount of Cu(NO_3_)_2_·3H_2_O (Sigma-Aldrich, Massachusetts, United States) precursor was dissolved in 40 mL of distilled water, in which 4.4 g of the pre-calcined SBA-15 support had been added under stirring at room temperature for few minutes. This mixture was then heated at 65 °C, under stirring, until the complete evaporation of the water. The resulting powder was dried overnight at 80 °C in an oven and then calcined in a tubular reactor at 600 °C for 5 h with a ramp of 2 °C/min under synthetic air flow (10 L/h). BaO was also wet-impregnated on the prepared CuO/SBA-15 powder by using Ba(CH_3_COO)_2_ (Sigma-Aldrich) as a precursor and following the same synthesis steps. Two CuO/BaO/SBA-15 adsorbents with the same CuO amount (6.5 wt.%) and different BaO loadings (5 and 24.5 wt.%) were prepared using this protocol.

### 2.2. Characterization Techniques

The chemical composition of the SO_2_ traps was determined by inductively coupled plasma-optical emission spectrometer (ICP-OES), using a HORIBA Jobin Yvon-Activa spectrometer, from powders previously dissolved in a mixture of inorganic acids (HNO_3_ and HF).

X-ray powder diffraction (XRD) was performed in reflection mode on a Bruker D8-A25 diffractometer (Cu Kα radiation, λ = 1.5406 Ǻ) equipped with a LYNXEYE XE-T linear detector. Wide-angle measurements were achieved for 2θ angle values in the 5–80° range, by steps of 0.04°. For low-angle measurements (2θ range from 0.45 to 7°, by steps of 0.01°, with a time of 118 s per step), a second XRD apparatus was used, the Bruker AXS diffractometer (Cu-Kα radiation, λ = 1.54184 Å) equipped with a LYNXEYE detector.

N_2_ physisorption analyses were performed on a Micromeritics Tristar 3000 apparatus. Prior to the measurements, the samples were first outgassed at 300 °C for 3 h under vacuum. SSAs were determined according to the BET (Brunauer–Emmett–Teller) method. Pore-size distributions were calculated by the BJH (Barrett–Joyner–Halenda) method on the desorption branch of the isotherms. 

The particle-size distribution was determined using laser diffraction granulometry (MALVERN MS3000). The particles were dispersed in ethanol with an ultrasonic treatment.

Scanning electron microscopy (SEM) micrographs were obtained on a ZEISS SUPRA-55 VP FEG microscope(Oberkochen, Germany). X-ray mappings were obtained from the EDX analyzer OXFORD X-Max 80 (Oxfordshire, United Kingdom) integrated on the same microscope. Images were collected with a voltage of 20 kV.

DRIFT spectroscopy was used to elucidate the sulfation phenomena on the SO_2_ powder trap. These measurements were carried out in a Thermo Scientific Nicolet iS50 spectrometer using a praying-mantis accessory (HARRICK high temperature reaction chamber) and a liquid-N_2_-cooled MCT detector with a resolution of 2 cm^−1^. The spectra were recorded from 1000 to 4000 cm^−1^. The background spectrum was collected on pure and dry KBr. DRIFTS spectra were collected on fresh and sulfated SO_2_ traps (i.e., traps that had been exposed to multiple NO-SO_2_ cycles) at 35, 200 and 500 °C under 50 mL/min of He. The reported spectra are the result of subtracting from the sulfated-trap spectra measured at each temperature that of the non-sulfated-trap (fresh adsorbent) used as a reference and also collected under He at the corresponding temperature. Finally, to verify the protection effect of the SO_2_ trap on the NOx sensor, the DRIFTS spectra of the SO_2_-poisoned sensor and the protected sensor were recorded at room temperature. The latter sensor is supposed to be protected by a SO_2_ trap. Note that the sensor was adapted to the reaction chamber of the DRIFT cell to favor the measurements directly on the sensor surface.

Thermogravimetric (TGA) analyses of sulfated trap were performed with a TGA Mettler-Toledo analyzer. The data were obtained in 1 L/h of air, from room temperature to 1300 °C, with a temperature ramp of 10 °C/min.

### 2.3. SO_2_ Adsorption and Regeneration Experiments

The SO_2_ adsorption capacity was investigated by cyclic adsorption and regeneration experiments in a fixed-bed reactor. Samples were introduced in a vertical U-shaped quartz reactor (12 mm inner diameter) fitted with a fritted quartz layer. Before the SO_2_ adsorption step, the adsorbent was heated at 500 °C (ramp of 10 °C/min from room temperature) under 20 vol.%O_2_/He for 1 h to eliminate surface impurities and the humidity. The adsorption experiments were performed at 500 °C for 1 h, using a gas mixture containing 75 ppm of SO_2_, 0.9 vol.% of O_2_ and 2 vol. % of H_2_O, balanced with He, to mimic the conditions encountered in a glass-melting furnace exhaust. The gas flow rate was fixed at 30 L/h and adjusted by BROOKS 5850 mass flow controllers. On-line SO_2_ analysis was continuously performed by a multi-gas FTIR analyzer (MKS 2030). The regeneration steps were performed right after the adsorption step, in the same experimental setup, during temperature-programmed desorption (TPD) experiments in He. TPD was performed from 500 to 700 °C at 10 °C/min in a He flow of 30 L/h, and the temperature was maintained at 700 °C for 15 min. After each cycle, the sample was cooled down to 500 °C in 20 vol.%O_2_/He to re-oxidize the Cu species.

The SO_2_ adsorption capacity of the adsorbent ($C_{\mathrm{ads}}^{\mathrm{TPD}\;}$expressed in moles of adsorbed SO_2_ per gram of adsorbent) was calculated by the integration of the TPD spectra, according to Equation (1):

(1) $$C_{\mathrm{ads}}^{\mathrm{TPD}\;}=\frac{F_{g\;}\int_{t0}^{\mathrm{tf}}C\;\mathrm{dt}}{10^{6\;}V_{m}m_{\mathrm{ads}}}$$

where Fg is the gas flow rate (in L/h); C is the SO_2_ outlet concentration (in ppm); t_0_ and t_f_ (in h) are, respectively, the starting and the ending time of the regeneration step; V_m_ is the molar volume (24.5 L/mol at 25 °C and 1 atmosphere) and m_ads_ is the mass of adsorbent (in g). All the SO_2_ adsorption–regeneration tests were performed using an adsorbent mass of 0.1 g. These adsorption–regeneration (TPD) cycles were repeated 3 times for each SO_2_ trap.

### 2.4. Experimental Setup for NOx Sensor Studies

**Figure 1:**
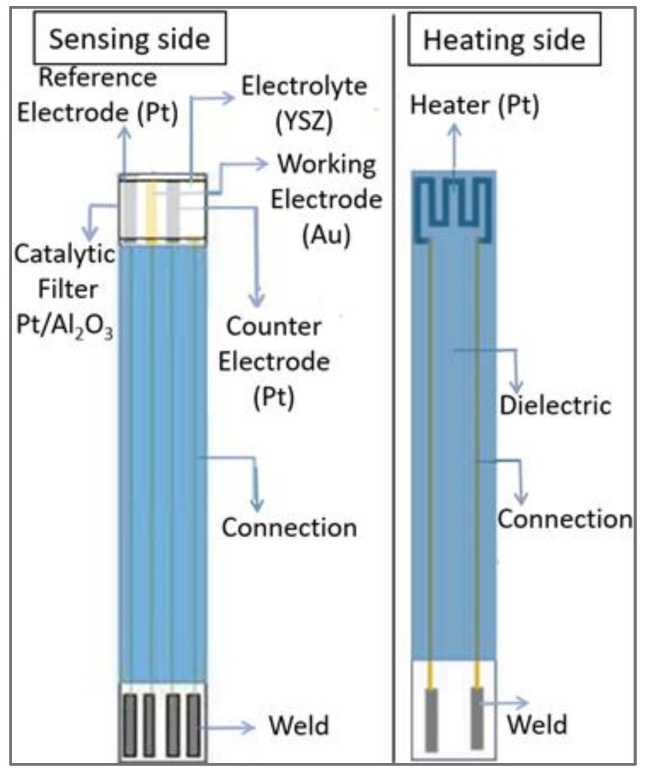
Scheme of the fabricated three-electrode sensor (Pt-Au-Pt) equipped with a catalytic filter (Pt/Al_2_O_3_).

The electrochemical planar sensor, which was composed of a YSZ porous layer and three metallic electrodes (Pt-Au-Pt), was elaborated by screen-printing (R23-Meteor 23″ semi-automatic screen-printer) on alumina substrates (2 cm × 5 cm), as described by Nematbakhsh et al. [57]. The electrolyte layer of YSZ (home-made ink) was first screen-printed and sintered at 1380 °C for 2 h. The electrodes were printed on a YSZ layer using commercial inks of Pt and Au, followed by 1 h sintering at 850 °C. A Pt heater was also printed on the reverse side of the alumina support to control the sensor temperature. The connectors, welding areas and protector dielectric film were deposited on both sides of the sensor. A catalytic-filter layer consisting of Pt (5 wt.%Pt, MERCK) dispersed in alumina was deposited over the sensing elements with a subsequent calcination process at 850 °C for 1 h. A schematic figure of the fabricated sensors is shown in Figure 1.

A measurement bench fully described by Nematbakhsh et al. [57] was used to study the responses of two sensors operating simultaneously in two separated cells. A polarization current of 50 nA was applied between the negatively polarized Au working electrode (WE) and the Pt counter-electrode (CE) to induce electrochemical reductions at the Au/YSZ interface. The sensor response (ΔV_RE_) was measured between the Pt reference electrode (RE) and WE. The responses to NO_2_, NO and SO_2_ in respective amounts of 100, 100 and 40 ppm were recorded at 510 °C in a base gas composed of 5 vol.% O_2_ and 1.5 vol.% H_2_O, balanced with N_2_. The total flow rate was fixed at 60 L/h, corresponding to 30 L/h per cell. The gas flow, regulated by mass flow controllers, passed first into a mixing chamber before being sent to the sensor cell. 

To study the ability of the prepared SO_2_ traps to protect the NOx sensor from sulfur poisoning, a “SO_2_ trap system” was integrated into the test bench and located upstream of the NOx sensor cell to adsorb the SO_2_ before it reaches the sensor. This system mainly consisted of a tubular furnace equipped with a vertical quartz flow-through reactor inside. The trap material was heated to 500 °C, as during the SO_2_ adsorption capacity tests. In this fixed-bed reactor, the used amount of each adsorbent was selected to optimize the adsorption capacity while maintaining a uniform gas flow without any pressure drop caused by the catalytic bed. According to the different particle arrangements in the reactor, catalyst weights were 0.8 g and 1.5 g for 6.5%CuO/5%BaO/SBA-15 and 6.5%CuO/24.5%BaO/SBA-15 traps, respectively. To assess the adsorption capacity of the SO_2_ traps and understand the sensors’ responses, FTIR gas analyzer (Gasmet DX 4015) was placed at the exits of the sensor cells for analyzing the SO_2_, NO and NO_2_ gases.

## 3. Results and Discussion

### 3.1. Physico-Chemical Characterizations of 6.5%CuO/5%BaO/SBA-15 and 6.5%CuO/24.5%BaO/SBA-15 Traps

Table 1 summarizes the chemical compositions, textural properties (determined from N_2_ physisorption) and particle sizes of the SBA-15 mesoporous silica calcined at 900 °C and of the two SO_2_ traps.

The actual percentages of CuO and BaO were in agreement with the theoretical ones (values reported in the sample nomenclatures), indicating that the synthesis method was appropriate. It is noteworthy that the small differences between these values could be considered acceptable, given the mass (5 g) of each prepared batch of the two adsorbents. 

**Figure 2:**
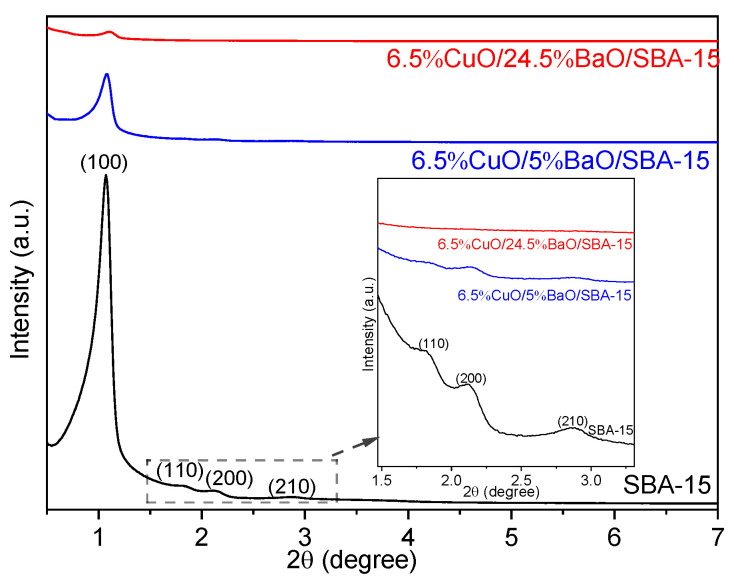
Low-angle XRD patterns of SBA-15 support (calcined at 900 °C) and CuO/BaO/SBA-15 adsorbents. For a better visibility, the diffraction patterns are shifted along the Y-axis. The insert corresponds to a zoom between 2θ = 1.5° and 2θ = 3.3°.

Figure 2 reports the low-angle XRD patterns of the SBA-15 support as well as the CuO/BaO/SBA-15 adsorbents. For SBA-15 support, the main reflections were located at 2θ = 1°, 1.8°, 2.1° and 2.8°, which correspond to the reflection planes (100), (110), (200) and (210). These planes confirm the ordered hexagonal mesoporous structure of SBA-15 with P6mm symmetry [58]. 

For both adsorbents, however, the intensity of the (100) reflection was strongly reduced, and the other reflections were not clearly observed, suggesting a loss in the crystallographic order of the mesoporous structure. Similar results were presented by Mathieu et al. [59], suggesting the formation of CuO and BaO metal oxides inside the mesopores of SBA-15 support after the calcination step. It must be emphasized that the diffraction peak (100) shows a slight shift towards higher angles, which is characteristic of a smaller lattice parameter and can probably be attributed to the successive calcination steps after the addition of both active phases [59].

**Figure 3:**
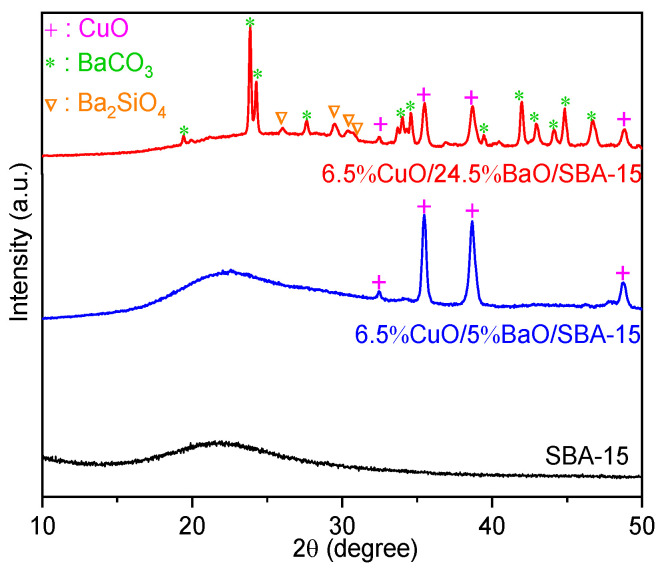
Wide-angle XRD patterns of SBA-15 support (calcined at 900 °C) and CuO/BaO/SBA-15 adsorbents. For a better visibility, the diffraction patterns are shifted along the Y-axis.

The corresponding wide-angle XRD patterns are reported in Figure 3. As expected, the SBA-15 support shows a very wide diffraction peak at 2θ = 20–30° without any other peaks, confirming its amorphous structure. After the addition of CuO and BaO, the XRD patterns of both adsorbents reveal the presence of crystalline CuO phase, suggesting the presence of large CuO nanoparticles after the calcination at 600 °C [48]. Moreover, for 6.5%CuO/24.5%BaO/SBA adsorbent, clear diffraction peaks corresponding to barium carbonate (BaCO_3_) formed in ambient air and barium silicate (Ba_2_SiO_4_) were detected. A high BaO loading seems to favor the solid-state reaction between BaO and the SBA support to form Ba_2_SiO_4._ This phase and Ba carbonate were not observed on the diffractogram of 6.5%CuO/5%BaO/SBA, most likely due to its lower Ba loading.

**Figure 4:**
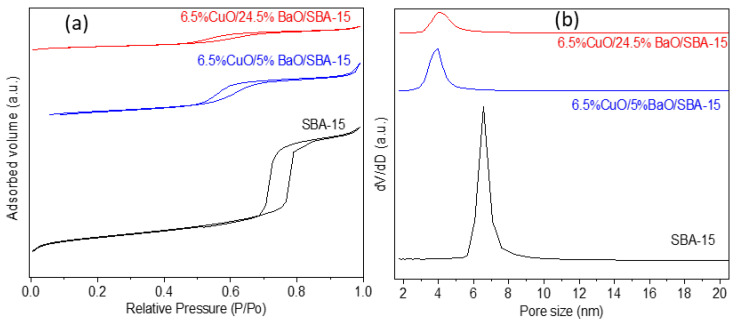
N_2_ physisorption isotherms (**a**) and the corresponding BJH pore-size distributions (**b**) of SBA-15 support (calcined at 900 °C) and CuO/BaO/SBA-15 adsorbents. For better visibility, the curves are shifted along the Y-axis.

The N_2_ physisorption isotherms and the corresponding BJH pore-size distributions of the SBA-15 support and the CuO/BaO/SBA-15 adsorbents are reported in Figure 4, and their textural properties are presented in Table 1. The SBA-15 support displays a type IV isotherm defined by parallel adsorption and desorption branches with a type H1 hysteresis loop in the relative pressure range P/P_0_ = 0.7–0.9. These profiles are characteristic of organized mesoporous materials with well-ordered cylindrical pores, according to the IUPAC classification [60]. However, for both CuO/BaO/SBA-15 adsorbents, the shape of the isotherm was modified, and the hysteresis showed a shift toward lower relative pressures. According to Berger et al. [61], this shift is probably due to the partial plugging of the pores with copper- or barium-based phases, in good agreement with wide-angle XRD patterns (Figure 3).

Moreover, the capillary condensation of both adsorbents is less steep, revealing a decrease in pore size (average pore sizes were 6.5 nm for SBA-15 and 4 nm for both adsorbents), as shown by Gaudin et al. [48]. The addition of CuO and BaO clearly affects the pore volume of the SBA-15 support, which decreases from 0.95 cm^3^/g for the support to 0.42 and 0.22 cm^3^/g for 6.5%CuO/5%BaO/SBA-15 and 6.5%CuO/24.5%BaO/SBA-15 adsorbents, respectively (Table 1). The same trend is also observed in SSAs, which decrease from 406 m^2^/g to 235 m^2^/g and 132 m^2^/g, respectively. These modifications in textural properties might be due to the blockage of the mesoporous support after the incorporation of the CuO and BaO active phases [49]. This effect is, as expected, more pronounced with a higher BaO loading. 

**Figure 5:**
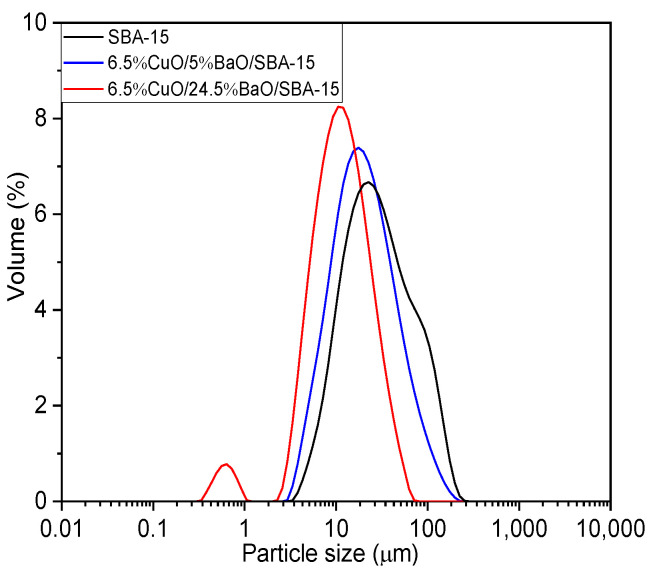
Particle-size distribution of SBA-15 support (calcined at 900 °C) and CuO/BaO/SBA-15 adsorbents, obtained by laser granulometry.

Figure 5 illustrates the particle-size distributions of SBA-15 and CuO/BaO/SBA-15 adsorbents, while Table 1 summarizes their median diameters (D50). The SBA-15 support calcined at 900 °C shows a unimodal size distribution with a median diameter D50 of 27 µm. However, a small bump is observed for diameters larger than 58 µm, which is probably due to particles being strongly agglomerated. After the incorporation of CuO and BaO, both adsorbents show a shift of particle-size distribution toward lower diameters.

The median diameters D50 are 19 µm and 11 µm for 6.5%CuO/5%BaO/SBA-15 and 6.5%CuO/24.5%BaO/SBA-15, respectively. This reduction in particle size after impregnation can be explained by the formation of small copper- or barium-based particles during the synthesis process. These particles can be detached from the support, leading to the decrease in the overall median diameter. Additionally, the metal oxide particles can diffuse into the pores of the SBA-15 support, leading to the formation of smaller particles due to confinement effects. Hence, these effects are more pronounced for the 6.5%CuO/24.5%BaO/SBA-15 adsorbent with a higher BaO loading, which shows a lower median diameter. It is noteworthy that the 6.5%CuO/5%BaO/SBA-15 adsorbent presents a unimodal size distribution, as does the SBA-15 support, while the 6.5%CuO/24.5%BaO/SBA-15 displays a small peak, at less than 1 µm, which could indicate the presence of a population of BaCO_3_ agglomerates. 

**Figure 6:**
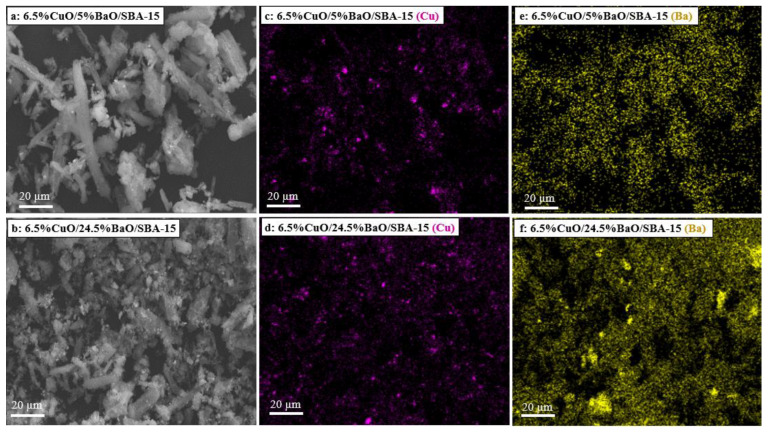
SEM micrographs (**a**,**b**) and corresponding X-ray mappings of copper (**c**,**d**) and barium (**e**,**f**) of 6.5%CuO/5%BaO/SBA-15 and 6.5%CuO/24.5%BaO/SBA-15 adsorbents, respectively.

The SEM micrographs and the corresponding X-ray mappings of adsorbents are shown in Figure 6. As expected from XRD results (Figure 3), the Cu-X-ray mappings of both adsorbents (Figure 6c,d) reveal the presence of large CuO agglomerates in the range of 2–3 µm that were also visible on the corresponding micrographs (Figure 6a,b), but a greater proportion of copper remains homogeneously distributed on the support. However, the Ba-X-ray mapping of the 6.5%CuO/5%BaO/SBA-15 adsorbent (Figure 6e) displays a homogeneous distribution of barium-based particles, whereas the 6.5%CuO/24.5%BaO/SBA-15 adsorbent (Figure 6f) shows relatively large micrometric agglomerates in the range of 5–10 µm. These results fit well with the XRD patterns (Figure 3), which exhibit strong BaCO_3_ and Ba_2_SiO_4_ peaks only for the 6.5%CuO/24.5%BaO/SBA-15 adsorbent with a higher BaO loading.

### 3.2. SO_2_ Adsorption Capacity Measurements

**Figure 7:**
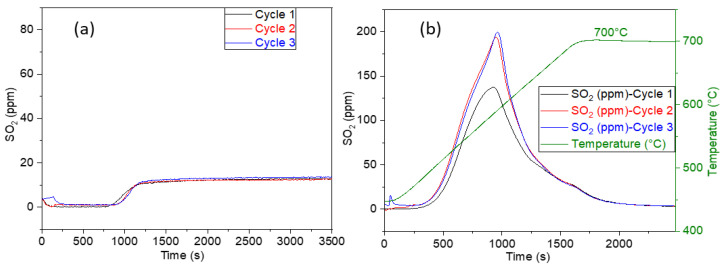
SO_2_ breakthrough (**a**) and TPD (**b**) curves of the 6.5%CuO/5%BaO/SBA-15 adsorbent submitted to three adsorption (75 ppm SO_2_, 0.9 vol.% O_2_, 2 vol.% H_2_O, balance He, 500 °C, 30 L/h) and regeneration (He, 700 °C, 30 L/h) cycles.

**Figure 8:**
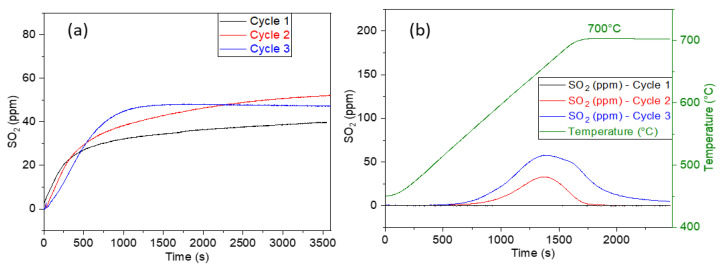
SO_2_ breakthrough (**a**) and TPD (**b**) curves of the 6.5%CuO/24.5%BaO/SBA-15 adsorbent submitted to three adsorption (75 ppm SO2, 0.9 vol.% O_2_, 2 vol.% H_2_O, balance He, 500 °C, 30 L/h) and regeneration (He, 700 °C, 30 L/h) cycles.

The CuO/BaO/SBA-15 adsorbents were subjected to successive SO_2_ adsorption–regeneration cycles in a fixed-bed reactor, as mentioned in the experimental design section. The SO_2_ breakthrough curves and TPD spectra obtained during multicycle experiments are reported in Figure 7 and Figure 8, respectively. The SO_2_ adsorption capacities, expressed in µmol_SO2_/g_ads_, are presented in Table 2.

For the first cycle, the 6.5%CuO/5%BaO/SBA-15 adsorbent shows a breakthrough at 15 min (Figure 7a) followed by an increase of the SO_2_ outlet concentration up to 13 ppm when 75 ppm of SO_2_ are injected for 1 h. This suggests that a fraction of SO_2_ is still adsorbed, and the saturation of the adsorbent surface has not been reached after 1 h on-stream. After the second and the third adsorption–regeneration cycles, similar shapes of the breakthrough curves were obtained, which indicates a good stability of the adsorbent with a great reproducibility of the adsorption step over cycles. From TPD curves (Figure 7b), the regeneration peaks occur at around 580 °C, which corresponds to the decomposition of copper sulfates, as described in the literature [48] and according with a previous test of a sulfated adsorbent containing only copper that showed a similar regeneration peak. The SO_2_ adsorption capacity, calculated by the integration of TPD curves (Figure 7b, Table 2), increases after the first cycle, from 312 up to around 400 µmol_SO2_/g_ads_, as confirmed by TPD curves (Figure 7b). As shown by Berger et al. [62], such a behavior could probably be assigned to some beneficial changes in the adsorbent porosity along the cycles, which would enable access to additional copper-based active sites.

The increase in the BaO loading modifies the SO_2_ adsorption properties (Figure 8a). No complete SO_2_ uptake was observed for the 6.5%CuO/24.5%BaO/SBA-15 over its cycles. After 1 h of sulfation under 75 ppm of SO_2_, the outlet concentration was around 40–50 ppm for the three cycles. The absence of a total SO_2_ adsorption period may be due to the gas flow having bypassed the adsorbent bed through preferential paths, as the bed height is 6 mm, and/or to possible intra-granular diffusional limitations which would have prevented the gas from accessing the pores of small grains. This phenomenon could limit the interaction between SO_2_ and the adsorbent, ultimately reducing the adsorption efficiency. In addition, the TPD curves of the first cycle (Figure 8b) do not show any desorption of SO_2,_ suggesting that sulfates formed during the first adsorption step are stable in He up to 700 °C. Despite the non-regeneration of the trap during the first TPD, the adsorbent continues to store SO_2_ during the second adsorption step at 500 °C while the second TPD shows a small SO_2_ desorption peak that further increases during the third TPD. This observation might be explained by the sulfation of some active sites that were bypassed by SO_2_ and therefore not sulfated during the first cycle. It should be noted that the regeneration peaks of this adsorbent occur at around 660 °C, whereas the other adsorbent, with lower BaO loading, shows desorption peaks at 580 °C (Figure 7b), which indicates the formation of different types of sulfates species. The SO_2_ storage capacity during the first several minutes increases with the number of cycles, suggesting an increase of the porosity of the adsorbent over cycles, limiting the intra-granular diffusional limitations.

As a partial conclusion, the 6.5%CuO/5%BaO/SBA-15 is more efficient than the 6.5%CuO/24.5%BaO/SBA-15 adsorbent for the adsorption of SO_2_. With the hypothesis that both adsorbents present the same CuO dispersion, the limited performance of the 6.5%CuO/24.5%BaO/SBA-15 adsorbent is probably related to a negative impact caused by the higher BaO loading. One possible explanation might be the formation of large barium-based particles, which have been previously identified by XRD and are visible by SEM-X-ray mapping analyses. In agreement with the findings of Centi et al. [38], the sulfation of such particles is a limiting process due to the slow bulk diffusion of SO_2_ molecules. Furthermore, as clearly highlighted by the N_2_ physisorption isotherms, these particles may cause a pore-blocking phenomenon, limiting the ability of SO_2_ to access active sites. It is also possible that during the second impregnation step, the higher BaO loading (24.5 wt.% vs. 5 wt.%) could have led to more blockage of the CuO sites, resulting in a significant reduction in the total number of accessible sites.

### 3.3. NOx Sensors’ Responses

As mentioned in the experimental section, a three-electrode NOx sensor (Pt-Au-Pt) equipped with a catalytic filter (Pt/Al_2_O_3_) was tested at 510 °C, with a polarization current of 50 nA between the Au working electrode and the Pt counter electrode. The sensor signal measurements were performed on an electronic card used to make galvanostatic measurements of the sensors, as described by Nematbakhsh et al. [57]. The galvanostat used allowed monitoring of the polarization current and the measurement of the potential between the working and reference electrodes.

To evaluate the capacity of the CuO/BaO/SBA-15 traps to protect our NOx sensor, the responses of this sensor to 100 ppm NO_2_, 100 ppm NO, and 40 ppm SO_2_ were recorded in the absence, and in the presence, of both SO_2_ traps heated to 500 °C (base gas: 5 vol.% O_2_, 1.5 vol.% H_2_O in N_2_). Before the injection of any gas (NOx or SO_2_), the sensor response was stabilized for 2 h on the polarization current.

**Figure 9:**
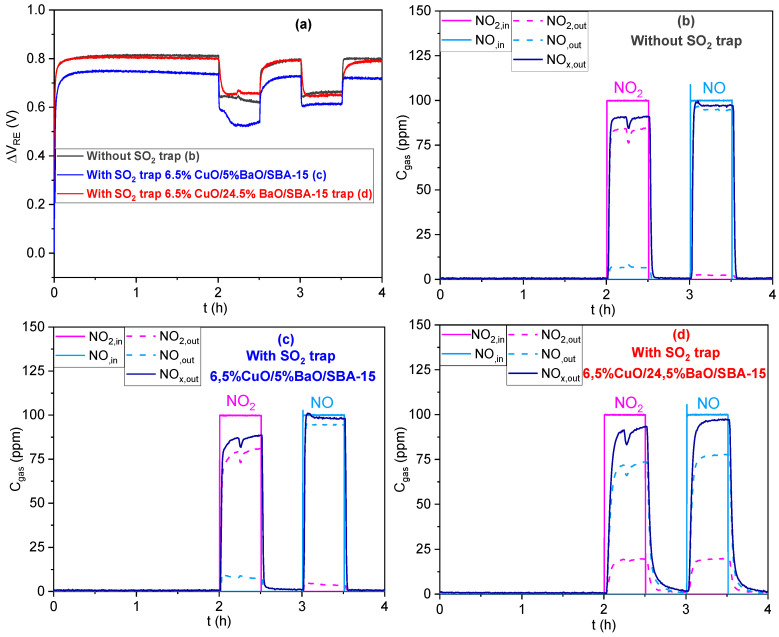
NOx sensor response (ΔV_RE_ = f (t)) to 100 ppm NO_2_ and 100 ppm NO (**a**) and the corresponding gas outlet concentrations (C_gas_= f (t)): (**b**) without SO_2_ trap; (**c**) with SO_2_ trap 6.5%CuO/5%BaO/SBA-15; and (**d**) with SO_2_ trap 6.5%CuO/24.5%BaO/SBA-15. Base gas: 5 vol.% O_2_, 1.5 vol.% H_2_O in N_2_. Solid lines illustrate the concentrations of NO and NO_2_ gases sent to the system and dashed lines correspond to gases analyzed at the outlet.

Figure 9a compares the sensor responses to NO_2_ and NO without, and with, a SO_2_ trap located upstream, and Figure 9b–d display the corresponding NOx outlet concentrations. The inlet gas is denoted as “in” and the outlet as “out”. The aim of these measurements is to investigate the interaction of the SO_2_ traps with NOx and the latter’s influence on the sensor response. The results in Figure 9a show that the sensor presents negative responses with similar amplitudes to NO_2_ and NO in the absence of the SO_2_ trap, as expected and reported by Gao et al. [22]. Indeed, when the sensor is equipped with a catalytic filter, the thermodynamic equilibrium of NO/NO_2_ is achieved, resulting in a response with the same amplitude and direction for either NO_2_ or NO.

Interestingly, the same behavior with very similar responses was obtained in the presence of either 6.5%CuO/5%BaO/SBA-15 or 6.5%CuO/24.5%BaO/SBA-15 traps up-stream of the sensor. The slight shift of the response towards lower ΔV_RE_ values observed with the 6.5%CuO/5%BaO/SBA-15 trap may be due to a small difference in the sensor temperature. 

To understand the sensor response during these three tests, the NOx outlet concentrations were analyzed. Without a SO_2_ trap (Figure 9b) and for an inlet NO_2_ concentration of 100 ppm, the gas analyzer displays a lower NO_2_ value (around 85 ppm) and also reveals the presence of NO (around 5 ppm) at the cell exit. Therefore, the total NOx concentration was 90 ppm for a NO_2_ set point of 100 ppm. The same results were reproduced, and may be due to a lower concentration in the NO_2_ cylinder or a small deviation of the mass flow controller. This may have no consequence for our results, since the total NOx concentration is relatively preserved and constant. For the NO gas, the outlet concentration fits with the set point of 100 ppm. It should be noted that the measured concentrations do not represent those at the sensor surface, where the NO/NO_2_ thermodynamic equilibrium should be reached due to the catalytic filter effect.

With the 6.5%CuO/5%BaO/SBA-15 trap (Figure 9c), the NOx concentrations at the outlet are very similar to those obtained without the trap (Figure 9b) for both NO_2_ and NO exposures. This result indicates that the 6.5%CuO/5%BaO/SBA-15 trap does not seem to significantly adsorb NOx, which is consistent with the specifications. It should be emphasized that the gas flow passes through the SO_2_ trap powder bed with a residence time, unlike its subsequent traversal through the catalytic filter of the sensor, which justifies the finding that the trap is the only factor responsible for gas composition variations. 

However, the 6.5%CuO/24.5%BaO/SBA-15 trap (Figure 9d) with a higher BaO loading presents an interaction with NO_2_ and NO. Indeed, for an inlet NO_2_ concentration of 100 ppm, only 20 ppm of NO_2_ was detected at the outlet, along with 73 ppm of NO, meaning that this trap can convert NO_2_ to NO. Nevertheless, the total NOx concentration remained relatively unchanged, compared to the test without the trap (Figure 9b). For an inlet NO concentration of 100 ppm, a less-pronounced conversion is obtained (78 ppm of NO and 20 ppm of NO_2_) and the same NOx concentration was detected at the outlet. Hence, this trap seems to reach the thermodynamic equilibrium NO/NO_2_, but without any significant NOx adsorption, since the total NOx concentration is still preserved. As previously mentioned in the introduction, BaO is known as a primary adsorbent material in NOx trap systems, with a much better ability to adsorb NO_2_ rather than NO [63]. However, according to Hess et al. [64], the adsorption of NOx on BaO at 500 °C is thermodynamically limited and the resulting nitrates/nitrites species are unstable, which can explain the very low NOx adsorption observed in our case. It is noteworthy that the mass used for this trap, which contained 24 wt.%BaO, is higher than the other one, which contained 5 wt.%BaO (1.5 g vs. 0.8 g), which could explain its more pronounced interaction with NOx.

Thus, with 100 ppm of either NO_2_ or NO in the inlet gas, similar NOx outlet concentrations were detected in the presence of both SO_2_ traps. This result can explain the similar responses of the NOx sensor observed in Figure 9a. Indeed, as shown by Gao et al. [22], the ΔV_RE_ value of a sensor equipped with a catalytic filter does not depend on the NO/NO_2_ reaching the sensor, but only depends on the total NOx concentration. It can be concluded that the presence of these traps does not influence the sensor response to NOx, which fits well with our objective.

**Figure 10:**
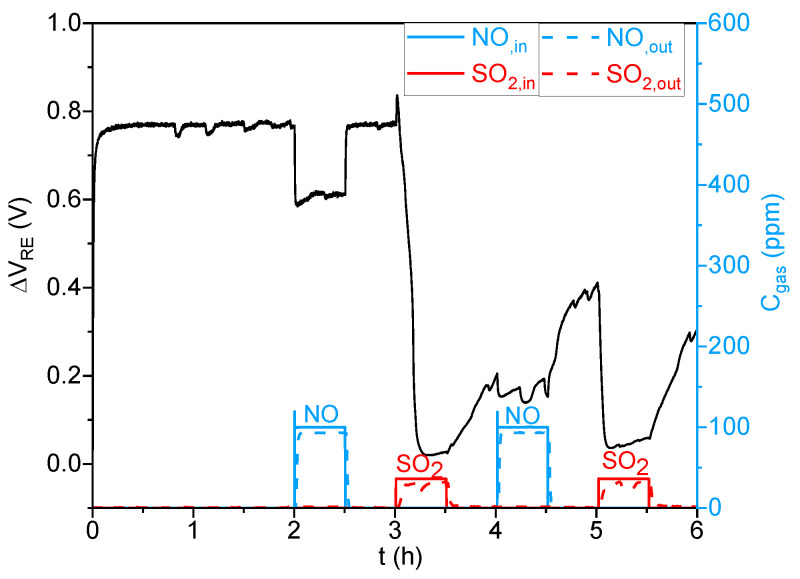
NOx sensor response (in black) (ΔV_RE_ = f (t)) to 100 ppm NO and 40 ppm SO_2_ in the absence of SO_2_ trap, with analysis of the gas outlet concentrations (C_gas_= f (t)).

To study the impact of SO_2_ on the sensors’ performance, the latter were successively subjected to 100 ppm of NO and 40 ppm of SO_2_ in the absence of a SO_2_ trap (Figure 10). As a result, the sensor showed a high and negative response to 40 ppm of SO_2_, with an important decrease in the baseline. Subsequently, its response to 100 ppm of NO was significantly reduced compared to that obtained before SO_2_ exposure. A negative and large response was observed again after the second SO_2_ exposure. Therefore, it can clearly be noticed that this sensor is significantly poisoned by SO_2_, becoming almost unable to respond to NO. 

**Figure 11:**
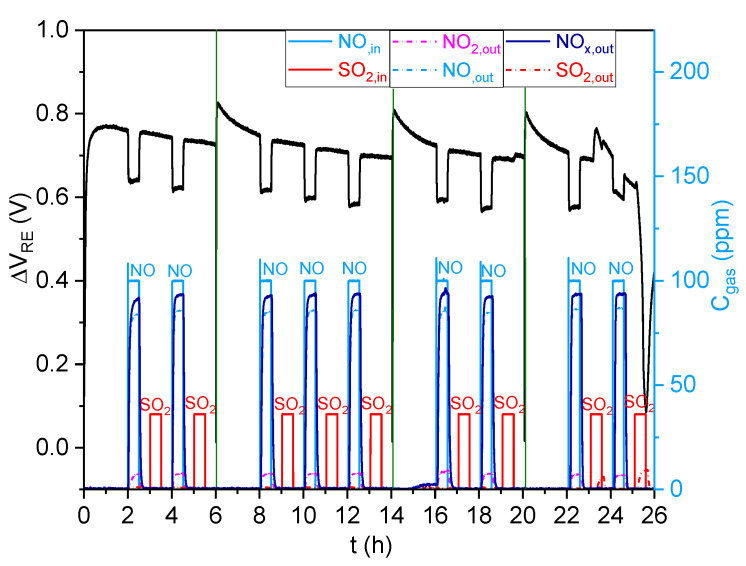
NOx sensor response (in black) (ΔV_RE_ = f (t)) to 100 ppm NO and 40 ppm SO_2_ in the presence of the 6.5%CuO/5%BaO/SBA-15 trap, with analysis of the gas outlet concentrations (C_gas_= f (t)).

**Figure 12:**
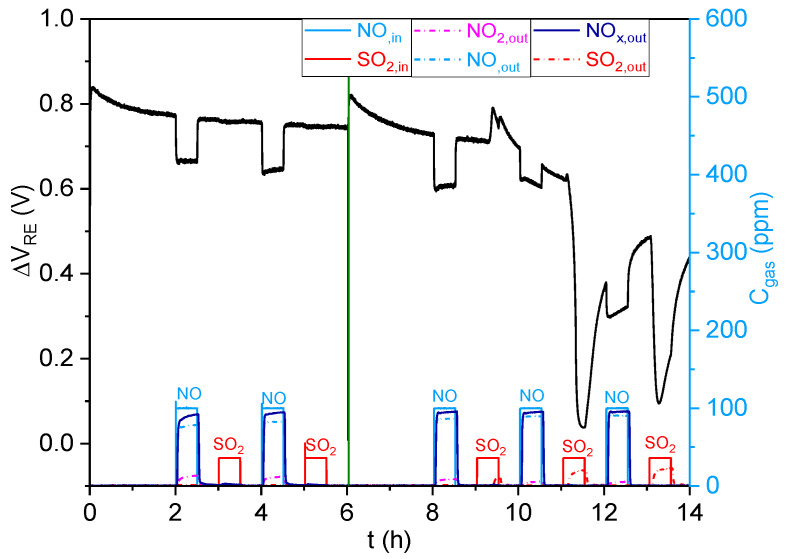
NOx sensor response (in black) (ΔV_RE_ = f (t)) to 100 ppm NO and 40 ppm SO_2_ in the presence of the 6.5%CuO/24.5%BaO/SBA-15 trap, with analysis of the gas outlet concentrations (C_gas_= f (t)).

To study the ability of the traps to protect the NOx sensor from sulfur poisoning, the response of this sensor to 100 ppm of NO and 40 ppm of SO_2_ was studied in the presence of the 6.5%CuO/5%BaO/SBA-15 trap (Figure 11) and the 6.5%CuO/24.5%BaO/SBA-15 trap (Figure 12), heated to 500 °C. The sensor was successively exposed to NO and SO_2_ until the surfaces of the traps reached saturation. The green lines are used to distinguish tests that were performed separately.

Figure 11 illustrates that during the first NO exposure, the 6.5%CuO/5%BaO/SBA-15 trap converted a fraction of NO to NO_2_, which is partially stored, resulting in a total NOx concentration between 82–90 ppm, instead of 100 ppm of NO in the inlet gas. As expected, the sensor showed a high negative response to the NOx gases. When exposed to SO_2_ for 30 min, the trap allowed a total SO_2_ adsorption, with no SO_2_ detected at the exit cell, resulting in a total protection of the NOx sensor from poisoning. Consequently, this trap exhibited a stable response to NO, compared with that obtained before the SO_2_ exposure. Until the eighth SO_2_ exposure for 30 min, the trap fully stored SO_2_ without any SO_2_ emissions, and the sensor response to NO was stable and reproducible. However, at the end of the eighth SO_2_ exposure (t = 23.5 h), some SO_2_ started to pass through the trap, causing the poisoning of the sensor. Therefore, the 6.5%CuO/5%BaO/SBA-15 trap can fully adsorb SO_2_ for up to 4 h under the experimental conditions used in this study (flow rate of 60 L/h containing 40 ppm of SO_2_ and m_ads_ = 0.8 g).

The 6.5%CuO/24.5%BaO/SBA-15 trap (Figure 12) exhibited a very low NO adsorption, with a NOx outlet concentration of 90 ppm. This trap allowed a total SO_2_ adsorption until the final minutes of the third SO_2_ exposure for 30 min, at which point the SO_2_ started to be detected at the outlet. At this stage, the sensor showed an unstable response to SO_2_, with a strong change in its baseline. Afterward, the sensor response to NO gradually decreased as the SO_2_ outlet concentration increased, until the sensor became almost completely unable to respond to NO. Thus, the 6.5%CuO/24.5%BaO/SBA-15 trap may be able to fully adsorb SO_2_ during a total period of 1.5 h (total flow rate of 60 L/h containing 40 ppm of SO_2_ and m_ads_ = 1.5 g).

Since the objective is to protect the NOx sensor from sulfur poisoning, we will only focus on the period of full adsorption of SO_2_ by the trap. Therefore, the SO_2_ adsorption capacities of both SO_2_ traps during the two previous tests (Figure 11 and Figure 12) were calculated based on the period where no SO_2_ emission was observed, and were denoted as $C_{\mathrm{ads}}^{\mathrm{Breakthrough}}$. The calculation is performed according to Equation (2):

(2) $$C_{\mathrm{ads}}^{\mathrm{Breakthrough}\;}=\frac{F_{g\;}C_{0\;}t}{10^{6\;}V_{m}m_{\mathrm{ads}}}$$

where F_g_ is the gas flow rate (60 L/h), C_0_ is the SO_2_ inlet concentration (40 ppm), t (in h) is the time period in which no SO_2_ emission was observed, V_m_ is the molar volume (equaling 24.5 L/mol at 25 °C and 1 atmosphere) and m_ads_ is the mass of adsorbent (in g). The calculated adsorption capacities are reported in Table 3. 

To meet our industrial requirements, a sufficient amount must be used for the SO_2_ trap to fully adsorb SO_2_ for 3 months (i.e., 2190 h) and therefore ensure a total protection of the NOx sensor. The proposed mixture to simulate the adsorption step corresponding to the glass-furnace exhaust contains 100 ppm of SO_2_, with a total flow rate of 10 L/h. Based on the SO_2_ adsorption capacities $C_{\mathrm{ads}}^{\mathrm{Breakthrough}\;}$, the mass of each trap required for 3 months of full SO_2_ adsorption under the specified conditions was calculated; the results are presented in Table 3.

As shown in Table 3, the 6.5%CuO/5%BaO/SBA-15 trap with the lower BaO loading presents a higher SO_2_ adsorption capacity (491 µmol_SO2_/g_ads_) than that of the 6.5%CuO/24.5%BaO/SBA-15 trap (98 µmol_SO2_/gads), in agreement with previous experiments. Therefore, the estimated mass values required to fully adsorb SO_2_ continuously during 3 months under the mentioned specifications are 182 g and 912 g for 6.5%CuO/5%BaO/SBA-15 and 6.5%CuO/24.5%BaO/SBA-15, respectively. It can be concluded that the 6.5%CuO/5%BaO/SBA-15 trap offers the best efficiency and can fully store SO_2_ and protect the NOx sensor for up to 3 months using a mass of 182 g, which appears consistent and feasible at the industrial scale.

### 3.4. Characterization of the SO_2_ Traps and of the NOx Sensor after Sulfation

**Figure 13:**
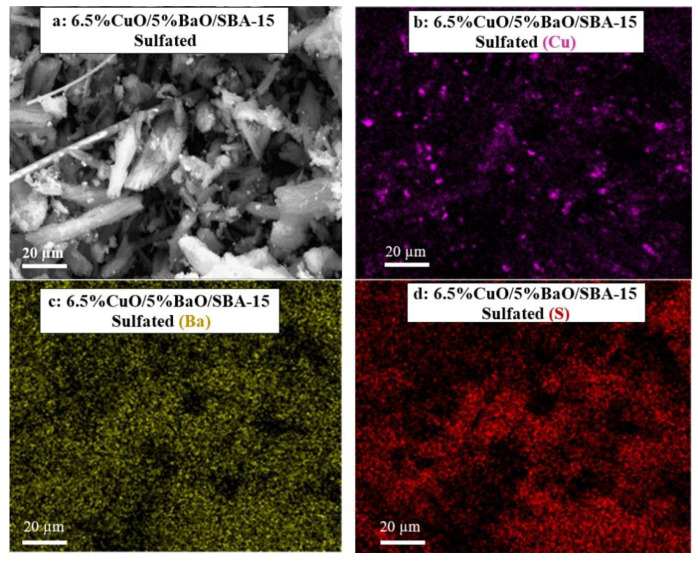
SEM micrograph (**a**) and X-ray mappings of copper (**b**), barium (**c**) and sulfur (**d**) of the 6.5%CuO/5%BaO/SBA-15 sulfated trap.

**Figure 14:**
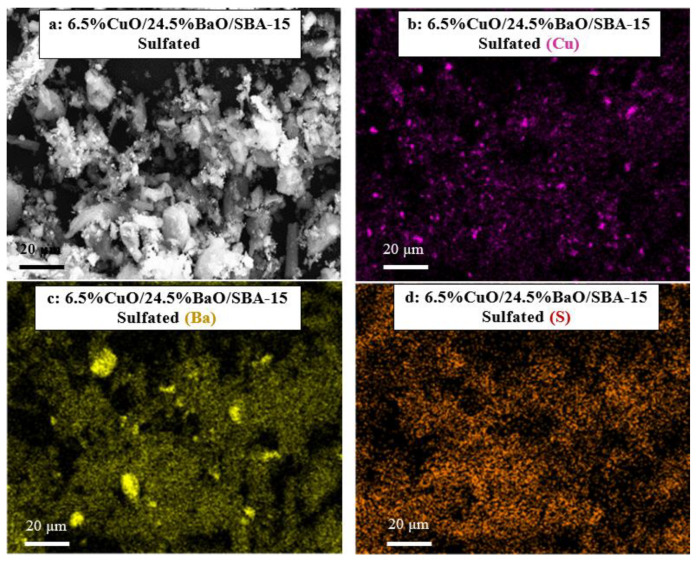
SEM micrograph (**a**) and X-ray mappings of copper (**b**), barium (**c**) and sulfur (**d**) of the 6.5%CuO/24.5%BaO/SBA-15 sulfated trap.

In order to highlight the sulfation and its effect on the active phases for SO_2_ adsorption, the two traps were characterized by SEM/X-ray mapping (Figure 13 and Figure 14, respectively) after their multiple NO-SO_2_ exposures, as reported in Figure 11 and Figure 12. 

The SEM micrograph (Figure 13a) and the Cu-X-ray mapping (Figure 13b) of the 6.5%CuO/5%BaO/SBA-15 trap reveal large CuO particles beside smaller ones, while the Ba-X-ray mapping (Figure 13c) shows a homogeneous distribution of Ba-based particles. The same results were observed with the non-sulfated trap (see Figure 6a,c,e) indicating that the sulfation does not significantly affect the dispersion of the active phases within the SBA-15 support. Interestingly, the S-X-ray mapping (Figure 13d) confirms the presence of sulfur species well distributed on the support, most likely copper or/and barium-based sulfates/sulfites. For the 6.5%CuO/24.5%BaO/SBA-15 trap (Figure 14), the distributions of CuO (Figure 14b) and BaO (Figure 14c) seem to be similar to those before sulfation (Figure 6d,f, respectively), since large CuO and BaO particles are still observed. Sulfur-based species (Figure 14d) are also identified for this type of trap, and probably formed in both CuO and BaO active phases. 

**Figure 15:**
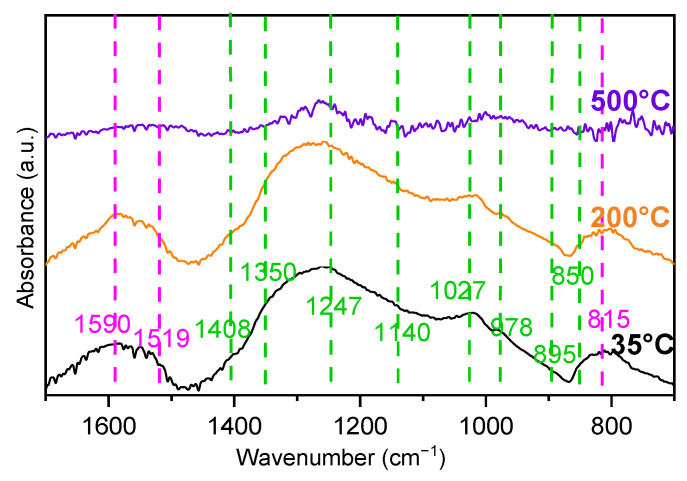
TPD-DRIFTS spectra for sulfated 6.5%CuO/5%BaO/SBA-15 SO_2_ trap (after subtracting the non-sulfated trap spectrum) collected at 35, 200 and 500 °C under He. Pink lines = bands associated with nitrates species, and green lines = bands associated with sulfate/sulfites species.

To identify the SO_2_-adsorbed species, the sulfated 6.5%CuO/5%BaO/SBA-15 trap (i.e., after the multiple NO-SO_2_ exposures, Figure 11), which is considered the most efficient, was also characterized by DRIFT spectrometry. Moreover, in order to identify in situ the possible regeneration of this trap, a TPD up to 500 °C in He was performed and also was followed by DRIFTS. Figure 15 shows the DRIFT spectra collected at 35, 200 and 500 °C under 50 mL/min of He. As previously mentioned in the experimental design section, the spectrum of the non-sulfated trap was taken as a reference and subtracted from that of the sulfated sample at the corresponding temperatures. 

At 35 °C, some broad bands were clearly observed at 978, 1027, 1140 and 1247 cm^−1^, which can be assigned to the bulk sulfate species adsorbed in BaO [28]. The presence of surface sulfate species was also corroborated by the bands observed in the region of 1340–1400 cm^−1^ [28,65]. Note that the two shoulders at 850 and 895 cm^−1^ are attributed to symmetric and asymmetric stretching of monodentate sulfite, respectively [66], suggesting the presence of some sulfites species on the sulfated trap after several cycles of NO-SO_2_ exposure. Moreover, in addition to the presence of sulfate/sulfite species, the bands at 815, 1519 and 1590 cm^−1^ indicate the presence of nitrate species. Indeed, the band at 815 cm^−1^ is attributed to bridging nitrate [66] or bulk nitrate adsorbed in the BaO phase [67], while the bands at 1519 and 1590 cm^−1^ are associated with monodentate nitrate [66,68] and chelating bidentate nitrate [67,68], respectively. However, the bands identified in the ranges of 950–1250 cm^−1^ and 1340–1400 cm^−1^, which were attributed to sulfates species, could be associated with some nitrates species (bands of surface nitrate species at 1000–1050 cm^−1^ and 1340–1410 cm^−1^ [66,67]). 

Therefore, it can be concluded that sulfates/sulfites species were formed during the sulfation of the 6.5%CuO/5%BaO/SBA-15 trap, as pointed out by these DRIFTS measurements. Moreover, these sulfates/sulfites species seem to be predominant on the trap surface, compared to nitrates, which suggests a limited adsorption of NO on the trap. However, the formation of some nitrate species, most likely on BaO sites, cannot be excluded.

At 200 °C, no significant changes in the spectrum were observed (Figure 15), suggesting that the adsorbed species are stable at this temperature. With the increase in the temperature to 500 °C, almost all the previously described bands disappeared, indicating that a non-negligible part of the adsorbed species was desorbed at this temperature. However, some small bands attributed to bulk sulfates species adsorbed on BaO sites were still present (978 and 1247 cm^−1^) suggesting that such species are stable at 500 °C. As reported by Mahzoul et al. [28], the decomposition of barium sulfates can occur at temperatures close to 1000 °C. Furthermore, a broad band in the region 1500–1600 cm^−1^ can be also observed, which corresponds to some nitrates that were not decomposed at 500 °C. Similar results have been reported in the literature [67,69], showing that nitrates species mainly decompose above 350 °C, but some of them may remain stable even at 500 °C. 

Based on these TPD-DRIFTS results, it can be concluded that the regeneration of this sulfated trap at 500 °C in He seems incomplete since some sulfates species are still present at this temperature. Thus, a higher temperature is required to completely regenerate this trap.

**Figure 16:**
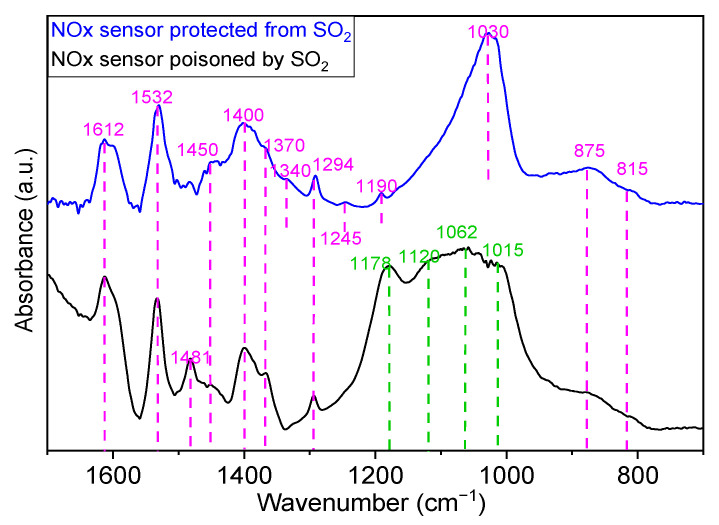
DRIFTS spectra of both the NOx sensor poisoned by SO_2_ and the NOx sensor protected from SO_2_, collected at room temperature. Pink lines = bands associated with nitrates/nitrites species, and green lines = bands associated with sulfates species.

Finally, to confirm the protection of the NOx sensor from sulfur poisoning in the presence of the 6.5%CuO/5%BaO/SBA-15 trap, the DRIFTS spectra of a sensor that was poisoned by SO_2_ was compared to one that was supposed to be protected by a SO_2_ trap (Figure 16). Indeed, the poisoned sensor is the one used is the test presented in Figure 11. This sensor was subjected to multiple NO-SO_2_ exposures in the presence of the 6.5%CuO/5%BaO/SBA-15 trap before being poisoned by SO_2_ not adsorbed by the trap. On the other hand, the so-called protected sensor was exposed for 30 min to NO and 30 min to SO_2_ in the presence of the 6.5%CuO/5%BaO/SBA-15 trap, which showed a total SO_2_ adsorption. As an assumption, this sensor is supposed to be fully protected from any sulfur poisoning, which will be identified by these DRIFTS experiments (Figure 16). As previously mentioned in the experimental design section, it is important to note that the IR radiation was focused on the sensing side of the sensor covered by the Pt/Al_2_O_3_ catalytic filter, and the spectra were collected at room temperature. The spectrum of a fresh sensor was taken as a reference and subtracted from those of the poisoned and protected sensors.

As shown in Figure 16, several bands are common for both poisoned and protected sensors. The positions of these bands and their corresponding assignments are as follows: 815 cm^−1^ and 875 cm^−1^ for bridging nitrates [66,70], 1294 cm^−1^ and 1532 cm^−1^ for monodentate nitrates [66,68,71], 1612 cm^−1^ for bridging nitrate associated with Pt [68] and 1370, and 1400 and 1450 cm^−1^ for monodentate nitrite in the region of 1375–1410 cm^−1^ [72]. However, the poisoned sensor shows an additional band at 1481 cm^−1^ attributed to monodentate nitrate [71]. In the same way, some other bands attributed to nitrates/nitrites species are clearly observed on the protected sensor: 1190 cm^−1^ for monodentate nitrite [70]; 1030 and 1245 cm^−1^ for chelating or bidentate nitrates, respectively [70,72]; and 1340 cm^−1^ for water-solvated nitrate [66]. The presence of these different nitrates/nitrites species on both poisoned and protected NOx sensors can be mainly attributed to the adsorption of NO on either Pt- or Al_2_O_3_-available sites of the catalytic filter. Based on the working principle of the sensor, one can note that NO is not stored by this filter, but rather undergoes an adsorption–desorption equilibrium during the experiment. Moreover, the DRIFT spectrum of the poisoned sensor shows a broad band in the region of 930–1250 cm^−1^ which can be deconvoluted in four bands at 1015, 1062, 1120 and 1178 cm^−1^. These bands could be related to bulk sulfates species, which confirms the poisoning of this sensor by SO_2_ and explains the degradation of its performance, as previously shown in Figure 11. It is noteworthy that none of these sulfate bands were detected on the protected sensor, confirming our assumption that the 6.5%CuO/5%BaO/SBA-15 trap was able to fully capture SO_2_, providing a total protection of the sensor from poisoning. In addition, it should be noted that the monodentate nitrites species observed at 1370 and 1400 cm^−1^ on both sensors (1375–1470 cm^−1^ region [72]) can overlap with surface sulfates species in the region of 1340–1400 cm^−1^ [65]. As these two bands seem to be more intense in the spectra of the poisoned sensor than the protected one, they could be attributed to surface sulfate species rather than nitrate species. Furthermore, the band at 1015 cm^−1^ observed on the surface of poisoned sensor and attributed to sulfate species can also overlap with surface nitrate species in the region of 1000–1050 cm^−1^ [66]. 

Based on DRIFTS measurements, it can therefore be concluded that surface sulfates/sulfites and nitrates species were formed on SO_2_ traps after several cycles of NO-SO_2_ exposures, the former being clearly predominant, which suggests a limited adsorption of NO on the trap. A partial regeneration of the adsorbent surface was also pointed out by these experiments. However, the temperature should be increased to ensure full SO_2_ trap regeneration. Finally, the protection role of the SO_2_ trap for the NOx sensor was confirmed, showing that most of the species observed on the surface of the protected sensor can be attributed to the N-containing adsorbed species, and without clear evidence of sulfates/sulfites species. These species were, however, clearly present in the DRIFTS spectrum of the poisoned sensor.

**Figure 17:**
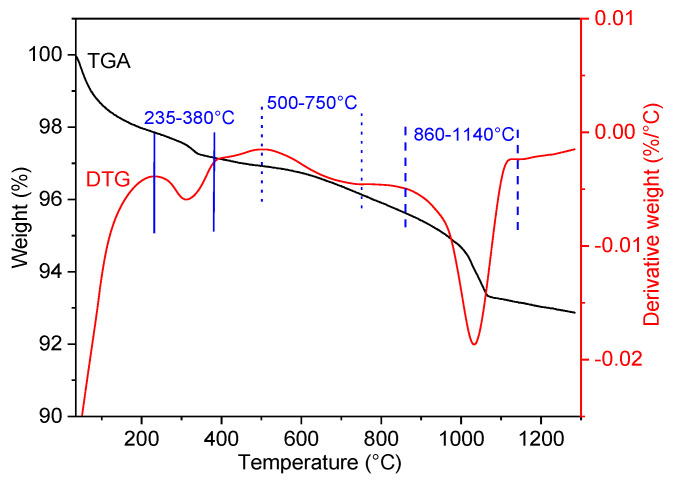
TGA-DTG analyses of the sulfated 6.5%CuO/5%BaO/SBA-15 trap.

Thermal analyses were performed on a sulfated 6.5%CuO/5%BaO/SBA-15 trap to identify the types of SO_2_-adsorbed species. This sulfation was performed at 500 °C and under 40 ppm of SO_2_ until the breakthrough, in the same base gas of previous tests (5 vol.% O_2_, 1.5 vol.% H_2_O in N_2_). The thermogravimetric (TGA) and derivative thermogravimetric (DTG) curves in the temperature range of 35–1300 °C are shown in Figure 17. A small weight loss is observed below 100 °C, which may correspond to the release of physically adsorbed water. Another weight loss, of about 0.75%, is detected between 230 to 400 °C. It may be due to the decomposition of copper carbonate into CuO, which occurs at temperatures of 230–300 °C, as previously reported by Shaheen et al. [73].

However, the main weight loss, of about 2.5%, is observed between 860 and 1140 °C and centered at 1030 °C. This weight loss could be due to the decomposition of barium sulfates or barium carbonates. Since no similar decomposition was observed in the thermal analysis of the fresh trap (results not shown), it can be deduced that this weight loss most likely corresponds to the decomposition of barium sulfates into BaO. Indeed, as shown by Mahzoul et al. [28], the barium sulfate may decompose at 1000 °C. Moreover, Yamazaki et al. [74] showed that such a decomposition could also occur at lower temperatures, around 800 °C, which matches well with our decomposition temperature range. It is important to note that a small weight loss can be observed in the temperature range of 500–750 °C, which may be attributed to the decomposition of copper sulfates to CuO, as found in the literature [48] and confirmed by our regeneration tests (see Figure 7b). Based on these thermal analysis results, it can be concluded that the barium sites of the 6.5%CuO/5%BaO/SBA-15 trap are probably more active toward SO_2_ adsorption than are the copper sites. However, BaO presents a limited catalytic activity for the oxidation of SO_2_ to SO_3_, which is essential for subsequent adsorption. Therefore, the presence of CuO in the catalyst is necessary to enable this oxidation while giving rise to more adsorption sites.

## 4. Conclusions

In order to protect the electrochemical NOx sensor from sulfur poisoning under the glass-furnace exhaust, two different CuO/BaO/SBA-15 adsorbents were synthesized and tested for trapping SO_2_ at 500 °C during cyclic adsorption–regeneration experiments. The challenge was to identify a material able to oxidize and trap SO_2_, and with a low affinity to NOx. The whole of the characterizations of these traps shows: (1) the formation of large micrometric CuO particles for both traps, (2) finely dispersed small Ba particles for the 6.5%CuO/5%BaO/SBA-15 and some BaCO_3_ micrometric agglomerates on 6.5%CuO/24.5%BaO/SBA-15, and (3) a more pronounced decrease in porous volume, specific surface area and mesoporous organization observed for the 6.5%CuO/24.5%BaO/SBA-15 trap with higher BaO loading.

The SO_2_ adsorption–regeneration cycles show that the trap performance strongly depends on the BaO loading. The 6.5%CuO/5%BaO/SBA-15 trap exhibits a better efficiency/stability compromise, with higher adsorption capacity than that of the 6.5%CuO/24.5%BaO/SBA-15 trap after the third cycle.

The NOx sensor response was studied in the presence of the “SO_2_ trap system” placed upstream the sensor. Both traps showed a very low NOx adsorption and only acted in the NO_2_-NO conversion, while preserving the total NOx concentrations. Therefore, the NOx sensor response was not impacted by the presence of these traps.

The response of this sensor was investigated during multiple NO-SO_2_ exposures in the absence of, and in the presence of, SO_2_ traps. In the absence of traps, the sensor was significantly poisoned by SO_2_, becoming almost unable to respond to NO. However, in the presence of the 6.5%CuO/5%BaO/SBA-15 trap, a total SO_2_ adsorption across 4 h (m_ads_ = 0.8 g) was observed, along with a very low NO adsorption. Consequently, the NOx sensor was completely protected from SO_2_ poisoning and showed a stable response to NO. The 6.5%CuO/24.5%BaO/SBA-15 trap provided a much shorter protection, lasting for only 1.5 h, despite a higher mass (m_ads_= 1.5 g) implemented. Therefore, the calculated SO_2_ adsorption capacity corresponding to the full adsorption of SO_2_ ${(C}_{\mathrm{ads}}^{\mathrm{Breakthrough}})\;$was approximately five times higher for the 6.5%CuO/5%BaO/SBA-15 than for 6.5%CuO/24.5%BaO/SBA-15.

In our experimental conditions, a mass of 182 g of the 6.5%CuO/5%BaO/SBA-15 trap would be sufficient to continuously adsorb SO_2_ during three months of operation and to protect the NOx sensor from sulfur poisoning without affecting its response to NOx. This finding represents a major advance, as previously the main challenge was the interference of NO adsorption with that of SO_2_ for copper-based adsorbents. Given the long-term stability of NOx sensors in harsh environments, which has been previously highlighted in the literature, and with the demonstrated performance of the SO_2_ trap throughout the performed tests, it can be concluded that the whole system exhibits robust stability in such challenging conditions.

Finally, the SO_2_-adsorbed species on the sulfated traps were successfully identified by SEM-X-ray mapping and DRIFTS measurements. The thermal analysis suggests that SO_2_ is predominantly adsorbed on the BaO sites rather than on the CuO sites of the trap.

Future investigation should focus on designing a container for the SO_2_ trap and integrating it into industrial setups, upstream of the NOx sensor in the existing system. This container should present a compacted design that permits the gas to pass through without obstruction while maintaining a sufficient residence time. Such a design could serve as a unit replaced at regular intervals (e.g., every 3 months) once the catalyst reaches saturation. 

## Figures and Tables

**Table 1:** Chemical compositions, textural properties and particle sizes of the SBA-15-based adsorbents.

Sample	SBA-15 ^a^	6.5%CuO/5%BaO/SBA-15	6.5%CuO/24.5%BaO/SBA-15
CuO content (wt.%) ^b^	-	6.6	6.0
BaO content (wt.%) ^b^	-	4.8	22.3
SSA (m^2^/g)	406	235	132
Total pore volume (cm^3^/g)	0.95	0.42	0.22
Pore size (nm)	6.5	4	4
D50 (µm) ^c^	27	19	11

^a ^Calcined at 900 °C; ^b^ Determined by ICP analysis. The relative uncertainty is estimated to 0.1%; ^c^ Median particle size as determined by laser diffraction granulometry.

**Table 2:** SO_2_ adsorption capacity expressed per g of adsorbent of the 6.5%CuO/5%BaO/SBA-15 and 6.5%CuO/24.5%BaO/SBA-15 traps along three adsorption–regeneration cycles.

Adsorbent	${\mathrm{SO}}_{2}\;\mathrm{Adsorption}\;\mathrm{Capacity}\;(C_{\mathrm{ads}}^{\mathrm{TPD}\;}$) ^a^ (µmol_SO2_/g_ads_)		
	Cycle 1	Cycle 2	Cycle 3
**6.5%CuO/5%BaO/SBA-15**	312	398	395
**6.5%CuO/24.5%BaO/SBA-15**	-	44	121

^a^ Calculated from the integration of the TPD curves.

**Table 3:** SO_2_ adsorption capacities and the corresponding m_ads_ required for 3 months of full SO_2_ adsorption under specified conditions, for both CuO/BaO/SBA-15 traps.

Adsorbent	$C_{\mathrm{ads}}^{\mathrm{Breakthrough}\;}$ (µmol_SO2_/g_ads_)	m_ads_ (g) Required for 3 Months of Full SO_2_ Adsorption ^a^
**6.5%CuO/5%BaO/SBA-15**	491	182
**6.5%CuO/24.5%BaO/SBA-15**	98	912

^a^ Considering 100 ppm of SO_2_ and a total flow rate of 10 L/h.

## Data Availability

The data that support the findings of this study are available from thecorresponding author upon reasonable request.
